# Role of endogenous and exogenous female sex hormones in arthritis and osteoporosis development in B10.Q-ncf1^*/* ^mice with collagen-induced chronic arthritis

**DOI:** 10.1186/1471-2474-11-284

**Published:** 2010-12-16

**Authors:** Caroline Jochems, Ulrika Islander, Malin Erlandsson, Cecilia Engdahl, Marie Lagerquist, Inger Gjertsson, Claes Ohlsson, Rikard Holmdahl, Hans Carlsten

**Affiliations:** 1 Department of Rheumatology and Inflammation Research, The Sahlgrenska Academy at Göteborg University, Göteborg, Sweden; 2 Center for Bone Research at the Sahlgrenska Academy (CBS), Göteborg, Sweden; 3 Medical Inflammation Research, MBB, Karolinska Institute, Stockholm, Sweden; 4 Medical Inflammation Research, Turku University, Turku, Finland

## Abstract

**Background:**

Collagen-induced arthritis (CIA) is an often-used murine model for human rheumatoid arthritis (RA). Earlier studies have shown potent anti-arthritic effects with the female sex hormone estradiol and the selective estrogen receptor modulator (SERM) raloxifene in CIA in DBA/1-mice. B10.Q-ncf1^*/*^mice are B10.Q mice with a mutated Ncf1 gene. In B10.Q-ncf1^*/*^mice, CIA develops as a chronic relapsing disease, which more accurately mimics human RA. We investigated the role of endogenous and exogenous sex steroids and raloxifene in the course of this model of chronic arthritis. We also examined whether treatment would prevent the development of inflammation-triggered generalized osteoporosis.

**Methods:**

Female B10.Q-ncf1^*/*^mice were sham-operated or ovariectomized, and CIA was induced. 22 days later, when 30% of the mice had developed arthritis, treatment with raloxifene, estradiol or vehicle was started, and the clinical disease was evaluated continuously. Treatment was continued until day 56 after immunization. At termination of the experiment (day 73), bone mineral density (BMD) was analyzed, paws were collected for histological examination, and sera were analyzed for markers of cartilage turnover and pro-inflammatory cytokines.

**Results:**

Raloxifene and estradiol treatment, as well as endogenous estrogen, decreased the frequency of arthritis, prevented joint destruction and countered generalized osteoporosis. These effects were associated with lower serum levels of the pro-inflammatory cytokine IL-6.

**Conclusions:**

This is the first study to show that raloxifene and estradiol can ameliorate established erosive arthritis and inflammation-triggered osteoporosis in this chronic arthritis model. We propose that treatment with raloxifene could be a beneficial addition to the treatment of postmenopausal RA.

## Background

Rheumatoid arthritis (RA) is a joint destructing autoimmune disease affecting 0.5-1% of the adult population [1,2]. The distribution between men and women is 1:3, with a peak incidence during menopause and in the post-partum period [3]. Several studies, including a population-based case-control study [4], have investigated whether the use of oral contraceptives could have an impact on the development of RA. Most of these studies found that current or ever use of oral contraceptives do have a protective effect (reviewed in [5]). The use of hormone replacement therapy (HRT) has been associated with some beneficial effects on disease activity [6-9]. For instance, a prospective two-year trial of 88 postmenopausal women with RA found that estrogen-containing HRT ameliorated clinical disease, retarded joint destruction, and increased bone mineral density (BMD) [6]. Estradiol-treatment of collagen-induced arthritis (CIA) in mice also suppressed disease progression [10,11], and blocking of the estrogen receptors enhanced the disease [12]. CIA is a model of human RA, and has been widely used to investigate disease mechanisms and treatments [13]. We have previously shown potent anti-arthritic effects of the selective estrogen receptor modulator (SERM) raloxifene in CIA in mice, when raloxifene was given as prophylaxis, therapy or in severe established disease [14,15]. Raloxifene hampered arthritis development, joint destruction and the development of generalized osteoporosis to the same degree as estradiol treatment. The rationale for using raloxifene instead of HRT is that estrogen treatment has been shown to increase the risk for cancer of the breast and uterus, as well as stroke, whereas raloxifene treatment does not have these side effects [16-18].

A polymorphism of the Ncf1 gene regulates the severity of arthritis in rats and mice, and has been shown to be caused by NADPH oxidase deficiency [19]. This results in a lower oxidative burst in macrophages, leading to spontaneous arthritis during the postpartum period, and to a more severe chronic relapsing collagen-induced arthritis disease in B10.Q mice with a mutated Ncf1 gene (B10.Q-ncf1^*/*^mice) [20,21]. The importance of reactive oxygen species in human RA was recently investigated in a Swedish case-control cohort consisting of 1842 RA cases and 1038 controls [22]. They found a genetic association between RA and the NADPH-oxidase complex, thus supporting the previous findings from animal models.

The role of endogenous and exogenous sex hormones in this chronic arthritis model has not previously been studied. We also wanted to investigate whether raloxifene would have a beneficial effect in this model. In addition we evaluated if treatment would prevent arthritis-induced osteoporosis, which is prominent in CIA [23] and postmenopausal RA [24,25], but has not previously been reported in arthritic B10.Q-ncf1^*/* ^mice.

## Methods

### Animals and experimental procedures

The ethical committee for animal experiments at Göteborg University approved this study. Female B10.Q-ncf1^*/* ^mice were generated as previously described [20]. Mice were electronically tagged and kept, 5 to 10 animals per cage, under standard environmental conditions, and fed standard laboratory chow and tap water *ad libitum*.

Ovariectomy and sham operations were performed at 7-19 weeks of age. Mice of different ages were mixed in each cage to avoid differences between the treatment groups. Ovaries were removed through a midline incision of the skin, and flank incisions of the peritoneum. The skin incision was closed with metallic clips. Sham-operated animals had their ovaries exposed but not removed. Surgery was performed after ketamine (PfizerAB, Täby, Sweden) and medetomidin (OrionPharma, Espoo, Finland) anesthesia. Carprofen (OrionPharma) was used post-operatively as a painkiller.

### Induction and evaluation of arthritis

2 weeks after ovariectomy CIA was induced by immunization with 100 μg of chicken type II collagen (CII) (Sigma, St Louis, MO, USA) dissolved in 0.1 M acetic acid and emulsified with an equal volume of incomplete Freund's adjuvant (Sigma) supplemented with 0.5 mg/ml *Mycobacterium tuberculosis *(Sigma). A total volume of 100 μl was injected intradermally at the base of the tail. Mice had already developed arthritis three weeks after immunization. Arthritis frequency was evaluated continuously until termination of the experiment. Scoring was performed in a blinded way without knowledge of the treatment groups and previous scores, as described previously [26], scoring 1 to 3 in each paw (maximum of 12 points per mouse) as follows: 1, swelling or erythema in one joint; 2, swelling or erythema in two joints; 3, severe swelling of the entire paw or ankylosis. The experiment was terminated 73 days after immunization.

### Treatment

**Figure 1:**
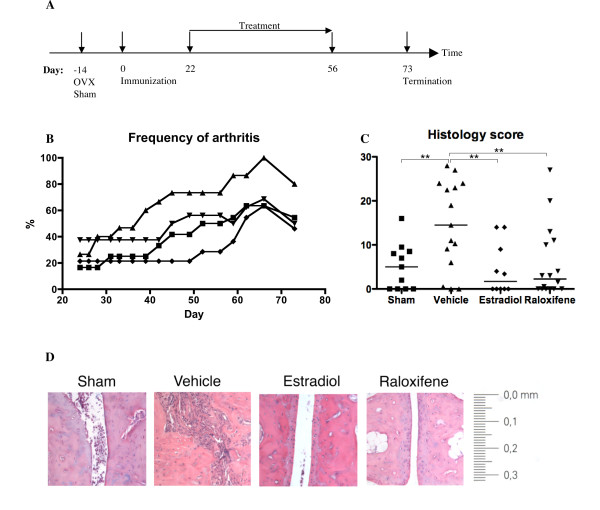
**Treatment with raloxifene or estradiol hampered arthritis development and joint destruction**. Female B10.Q-ncf1^*/* ^mice were sham-operated or ovariectomized, and immunized with collagen II and Freund's incomplete adjuvant supplemented with mycobacteria to induce CIA. Three weeks later treatment was started with raloxifene (60 μg/day; n = 15) (turned triangles), estradiol (1 μg/day; n = 11) (prisms), or vehicle control (Miglyol812) for sham-operated mice (n = 11; squares) and OVX controls (n = 15; triangles). Treatment was continued until day 56 after immunization, and the experiment was terminated on day 73. **A**: Diagram of the experimental timeline. OVX, ovariectomy; sham, sham operation; immunization with collagen II and Freund's complete adjuvant on day 0. **B**: Frequency of arthritis. Treatment with raloxifene in OVX mice delayed the onset and the frequency of arthritis compared to vehicle-treated OVX controls (*P *< 0.05 for the entire experiment). The presence of endogenous hormones (sham-operated mice) or estradiol treatment also delayed the onset of arthritis and decreased the frequency (*P *< 0.001 and *P *< 0.001, respectively). There was a significant difference between estradiol treatment and raloxifene treatment (*P *< 0.001). Kruskall-Wallis test with post hoc comparison was used. **C**: Histopathologic destruction scores of paw sections. Scores were evaluated in a blinded manner, with the proximal part of each paw graded 0-4, and the distal part graded 0-3, yielding a maximum score of 28 per mouse, as follows: 1 = synovial hypertrophy; 2 = pannus, erosions of cartilage and bone; 3 = severe erosions of cartilage and bone; 4 = complete ankylosis. The scatter plot shows the scores of individual mice, and lines show the median in each group. Kruskall-Wallis test with post hoc comparison was used, **P < 0.01. **D**: Representative images of paw tissue sections, revealing treatment effects on histologic features in each group.

Mice were given subcutaneous injections at different locations 5 days per week of the raloxifene analogue LY117018 (generous gift from Eli Lilly, Indianapolis, IN, USA) (60 μg/mouse/day) or 17β-estradiol-3-benzoate (E2) (Sigma) (1.0 μg/mouse/day) dissolved in Miglyol812 (OmyaPeralta GmbH, Hamburg, Germany). Miglyol812 is a very pure neutral oil from fractionated plant fatty acids C_8 _and C_10_. OVX and sham control mice received Miglyol812 (100 μl/mouse/day). The dosages of raloxifene and E2 have previously been shown to equally well prevent osteoporosis in mice [27-29]. Treatment with raloxifene, estradiol or vehicle five days per week was started therapeutically, 3 weeks after immunization, when approximately 30% of the mice had developed arthritis. Treatment was ended on day 56 after immunization, and the mice were followed until day 73, when they were sacrificed (Figure 1A).

LY117018 differs from raloxifene at only one site on the molecule, with a pyrrolidine ring on the basic side chain instead of a piperidine ring. This small difference does not affect its biological properties [30].

### Tissue collection and histologic examination

At termination of the experiment, mice were anaesthetized for blood withdrawal, and then killed by cervical dislocation. Sera were individually collected and stored at -20°C until used. One femur was placed in formaldehyde for 24 hours, and then in 70% ethanol, for analysis of bone mineral density. Paws were fixated in paraformaldehyde, decalcified for 30 hours in Parengy's decalcification solution (Histolab Products AB, Gothenburg, Sweden), and dehydrated over night in a vacuum infiltrator processor (Tissue-Tek V.I.P., Sakura Finetechnical Co. Ltd., Tokyo, Japan). After being embedded in paraffin they were sectioned in 4 μm sections on a Leica RM2255 (Leica Instruments GmbH, Nussloch, Germany). The sections were deparaffinated and stained in Mayer's hematoxylin followed by eosin. The sections were coded before examination. In sections from each animal, the proximal parts of all four paws were graded separately on a scale of 0-4, and the distal part was graded 0-3. This yielded a maximum histopathological destruction score of 28 points per mouse, assessed as follows: 1 = synovial hypertrophy, 2 = pannus, discrete erosions of cartilage and bone, 3 = severe erosions of cartilage and bone, 4 = complete ankylosis.

### Assessment of BMD

One femur was subjected to a peripheral quantitative computed tomography (pQCT) scan with a Stratec pQCT XCT Research M, software version 5.4B (Norland, Fort Atkinson, WI) at a resolution of 70 μm, as described previously [31]. Trabecular BMD was determined with a metaphyseal scan at a point 3% of the length of the femur from the growth plate. The inner 45% of the area was defined as the trabecular bone compartment. Cortical BMD was determined with a mid-diaphyseal scan.

### Identification of serologic markers of cartilage remodeling

As a marker of cartilage destruction, serum levels of COMP (cartilage oligomeric matrix protein) were determined with an Animal COMP^® ^ELISA kit (AnaMar Medical AB, Uppsala, Sweden).

### Serum IL-6 bioassay

A bioassay using the cell line B13.29, subclone B9 (which is dependent on IL-6 for growth) was used to measure serum levels of IL-6, as described previously [32,33].

### Statistical analysis

For statistical evaluation, the Kruskall-Wallis test followed by a post hoc test were used for comparisons between all groups in each experiment. A *P *value ≤ 0.05 was considered significant.

## Results

### Endogenous and exogenous estrogen as well as raloxifene treatment hampered arthritis in B10.Q-ncf1^*/* ^mice and protected joints from destruction

To examine the anti-arthritic properties of sex steroids, ovariectomized female B10.Q-ncf1^*/* ^mice were treated therapeutically from day 22 until day 56 post immunization, with either raloxifene (60 μg/day), estradiol (1.0 μg/day) or vehicle (Miglyol 812). Sham operated controls received Miglyol 812. Arthritis development was evaluated continuously. Treatment was ended on day 56 to investigate how this would affect the disease.

The cages of mice were randomly divided into the different treatment groups in a blinded way, without knowledge of the arthritic score. As shown in Figure 1B, the raloxifene treatment group had a frequency of arthritis of 40% at the start of treatment, whereas the other groups only displayed 20% of mice with arthritis. In spite of this difference, our results clearly show that raloxifene treatment resulted in a slower onset of arthritis and a lower frequency of disease, compared to vehicle treated mice (*P *< 0.05 for the entire experiment). We also found a significantly slower onset of disease in sham-operated mice and in mice treated with estradiol compared to vehicle controls (*P *< 0.001 and *P *< 0.001, respectively), and between estradiol treatment and raloxifene treatment (*P *< 0.001).

Histological examination of sections from the paws revealed severe destruction of the joints in the vehicle treated controls. In contrast, examination of joints from raloxifene and estradiol treated mice revealed a lower destruction score (*P *< 0.01) (Figure 1C and 1D). Similarly, endogenous estrogen (sham-operated mice) protected the joints from destruction compared to OVX controls (*P *< 0.01).

### Endogenous and exogenous estrogen, and raloxifene treatment counteracted osteoporosis in arthritic mice

**Figure 2:**
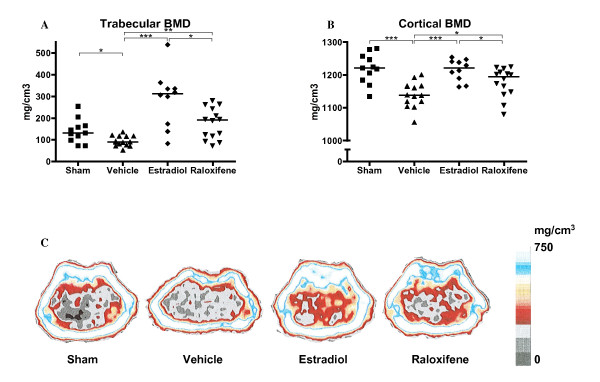
**Treatment with raloxifene or estradiol protected arthritic mice from osteoporosis**. Scatter plots showing the trabecular bone mineral density (BMD) (**A**) and the cortical BMD (**B**), measured with peripheral quantitative computer tomography (pQCT) in mice with CIA treated with raloxifene (60 μg/day), E2 (1 μg/day) or vehicle Miglyol812 (100 μl/day), *n *= 11-15 in each group. Lines represent medians. Kruskall-Wallis test with post hoc comparison was used, *P < 0.05, **P < 0.01, ***P < 0.001. **C: **Representative pQCT scans of cross-sections of the femur, showing the bone mineral density (BMD). The pictures show one representative mouse in each treatment group. The bar shows the density of the bone, from 0 (black) to >750 mg/cm^3 ^(white).

All mice in this experiment displayed low bone mineral density (BMD) compared to historic non-arthritic controls [23], where we have previously found the trabecular and cortical BMD to be 275 and 1270 mg/cm^3^, respectively. Vehicle treated OVX mice developed severe loss of trabecular and cortical BMD compared to sham-operated mice (*P *< 0.05 and *P *< 0.001), indicating a protective effect of endogenous sex hormones. In contrast, treatment with raloxifene and estradiol resulted in preserved trabecular and cortical BMD, with estradiol treated mice displaying the highest BMD (*P *< 0.05 and *P *< 0.001, respectively) (Figure 2).

### Endogenous and exogenous estrogen, and raloxifene treatment lowered the serum levels of IL-6

**Figure 3:**
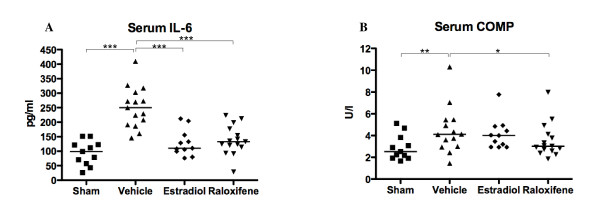
**Treatment with raloxifene decreased the serum levels of IL-6 and COMP**. Scatter plots showing the serum levels of IL-6 (**A**) and cartilage oligomeric matrix protein (COMP) (**B**) in mice with CIA treated with raloxifene (60 μg/day), E2 (1 μg/day) or vehicle Miglyol812 (100 μl/day). *n *= 11-15 in each group. Lines represent medians. Kruskall-Wallis test with post hoc comparison was used, *P < 0.05, **P < 0.01, ***P < 0.001.

Increased serum IL-6 in arthritis is a marker of the general inflammatory disease. The serum levels of IL-6 were significantly lower (*P *< 0.001) in mice treated with raloxifene or estradiol, and in sham operated mice, than in vehicle controls (Figure 3A).

### Endogenous estrogen and raloxifene treatment decreased cartilage resorption

The serum levels of COMP (cartilage oligomeric matrix protein) are elevated in arthritis due to increased destruction of joint cartilage. Treatment with raloxifene significantly decreased cartilage destruction, as indicated by lower serum COMP levels, compared to vehicle treated mice (Figure 3B). Interestingly, sham-operated mice also had lower levels of COMP, whereas estradiol-treated mice displayed similar levels as vehicle-treated controls.

### Raloxifene did not affect uterine weight

**Figure 4:**
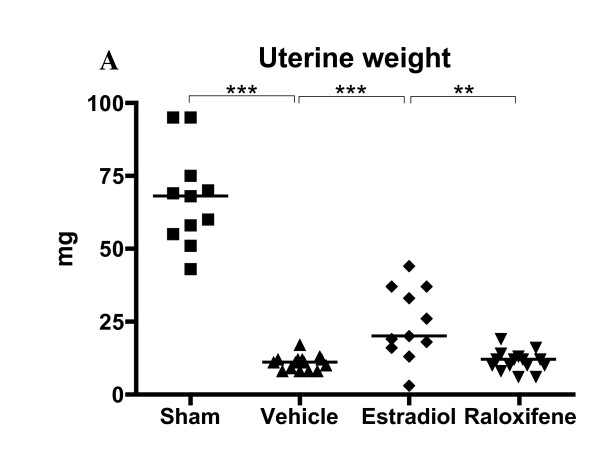
**Treatment with raloxifene did not increase uterine weight**. Scatter plots showing the uterine weights in mice with CIA treated with raloxifene (60 μg/day), E2 (1 μg/day) or vehicle Miglyol812 (100 μl/day) from day 22 to day 56 after immunization, and terminated on day 73, *n *= 11-15 in each group. Lines represent medians. Kruskall-Wallis test with post hoc comparison was used, **P < 0.01, ***P < 0.001.

Treatment of the mice was ended on day 56 post immunization, and the experiment was terminated on day 73. As shown in Figure 4, treatment with estradiol resulted in partly preserved increased uterine weight compared with OVX mice, even 2.5 weeks after the last injection, whereas raloxifene did not. This may be due to the cessation of treatment 2.5 weeks before termination of the experiment. Previous studies have found differential effects of raloxifene in uterine tissue. We have previously found a slight proliferative effect in mice treated for 2.5-3 weeks [28,34], whereas in another study mice were treated with a higher dose for 5 days, and they found no proliferative effect [35]. No increase in uterine weight was found in rats [36-38]. Most importantly, raloxifene was found to have antiproliferative and proapoptotic effects in women [39].

## Discussion

This is the first study to evaluate the impact of endogenous ovarian hormones, as well as exogenous estradiol and raloxifene, in the chronic arthritis model of B10.Q-ncf1^*/* ^mice. This model may more accurately relay the pathophysiologic process of RA than the widely used CIA model in DBA/1 mice, since it develops into a chronic active disease course [20]. We clearly show that the presence of ovarian hormones protects from development of arthritis and joint destruction. Interestingly, treatment with a physiological dose of estradiol or the selective estrogen receptor modulator raloxifene was equally efficient, although the treatment was given to already arthritic mice from day 22 to day 56 after collagen II immunization. The serum levels of COMP were significantly decreased in the raloxifene treated mice and mice with intact endogenous hormones compared to vehicle controls. This indicates a lower degree of on-going cartilage destruction. COMP was not decreased in the estradiol treated mice, although this group also displayed significantly less cartilage destruction. This finding is in accordance with the results in a recent study of CIA in rats demonstrating that estradiol exerts collagen type II and cartilage protective properties [40].

Women are more prone to develop RA than men, and the female peak incidence coincides with menopause, indicating that endogenous female sex hormones like estradiol have protective properties. Thus, we early proposed that treatment with estradiol would have disease-retarding effects in postmenopausal RA. The rationale for investigating the possible impact of raloxifene in this model is that previous studies have shown ameliorating effects of hormone replacement therapy containing estradiol in women with postmenopausal RA, but long-term therapy is no longer recommended due to the risk of side effects. We have previously shown, in the murine CIA model, that raloxifene treatment prophylactically or as therapy, or even when initiated in established disease, had a great ameliorating effect on arthritis, as well as on joint destruction and arthritis-induced osteoporosis [14,15].

Raloxifene binds with high affinity to estrogen receptor (ER) α, and acts as an estrogen agonist on bone tissue and blood lipids, but as an antagonist on breast and reproductive tissues [41]. We have recently shown that signaling through ERα (using propylpyrazoletriol) dramatically decreased the frequency and severity of arthritis, and prevented osteoporosis development, in CIA in DBA/1 mice, whereas signaling through the other estrogen receptors (ERβ and GPR30) did not affect arthritic disease or bone loss [42]. Raloxifene acts mainly via ERα, and its agonistic effects on this receptor were clearly seen in the current study, with the same anti-arthritic and anti-osteoporotic effects as estradiol.

We also recently showed that raloxifene activates the estrogen response element to the same extent as estradiol in bone, uterus and thymus, thus indicating that indeed the effects are mediated via the nuclear estrogen receptors [34]. Raloxifene has previously been shown to have a weak proliferative effect on mouse uterus, whereas estradiol displays a strong proliferative effect [28]. In the present study we found a lingering increase in uterus weight in mice treated with estradiol even though treatment had been halted 17 days before termination. No increase compared to vehicle treated mice was seen in the raloxifene treated group at termination, which may be due to the absence of treatment for the last 17 days of the experiment.

The serum levels of IL-6 were reduced in mice with intact endogenous sex hormones, or treated with estradiol or raloxifene. IL-6 targeting therapy is now being used to treat autoimmune diseases, including RA, and shows promising results with a significant clinical response and amelioration of joint damage (reviewed in [43]). In addition, it was recently shown that blockade of the IL-6 receptor both *in vivo *and *in vitro *directly affected osteoclast formation, suggesting a direct bone-sparing effect that is independent of the anti-inflammatory effects of anti-IL-6 treatment [44]. Thus, the lowering of serum IL-6 may be one mechanism by which raloxifene and estradiol exert their anti-osteoporotic and anti-arthritic effects.

## Conclusions

Based on the results of this study in the chronic arthritis model and our previous results in the more acute CIA model, we suggest using raloxifene treatment at the dose already approved for the treatment of osteoporosis (60 mg/day) as an addition to the standard treatment of postmenopausal RA. This could be a beneficial adjuvant with benefits on both joint damage and osteoporosis, and without the side effects of hormone replacement therapy with estradiol. A clinical trial addressing this issue is planned.

## Abbreviations

BMD: bone mineral density; CIA: collagen-induced arthritis; CII: type II collagen; COMP: cartilage oligomeric matrix protein; E2: estradiol; ELISA: enzyme-linked immunosorbent assay; ER: estrogen receptor; HRT: hormone replacement therapy; IL: interleukin; OVX: ovariectomy; pQCT: peripheral quantitative computed tomography; RA: rheumatoid arthritis; SERM: selective estrogen receptor modulator.

## Competing interests

The authors declare that they have no competing interests.

## Authors' contributions

HC, RH, CO and IG participated in study design, interpretation of data and manuscript preparation. UI aided with analysis of data and statistical analysis. ME, CE and ML aided with acquisition of data. The study was performed mainly by CJ. All authors read and approved the final manuscript

## Pre-publication history

The pre-publication history for this paper can be accessed here:

http://www.biomedcentral.com/1471-2474/11/284/prepub
